# Overcoming the Complexity of Total Laparoscopic Hysterectomy and Bilateral Salpingo-Oophorectomy in a Patient With Uterine Didelphys and Dual Endometrial Pathology: A Surgical Approach in a Complex Anatomical Variant

**DOI:** 10.7759/cureus.95107

**Published:** 2025-10-21

**Authors:** Mohamed Hemdan, Hani Shuheibar, Mohamed Alosta, Hassan Idris

**Affiliations:** 1 Obstetrics and Gynaecology, Manchester University NHS Foundation Trust, Manchester, GBR

**Keywords:** atypical endometrial hyperplasia, complex pelvic anatomy, congenital uterine malformation, laparoscopic hysterectomy bilateral salpingo-oophorectomy, müllerian anomaly, uterine didelphys

## Abstract

Uterine didelphys is a rare Müllerian anomaly occurring in approximately 0.1-0.5% of women. It is characterised by duplicated uterine and cervical structures and presents significant diagnostic and surgical challenges. The coexistence of atypical endometrial hyperplasia, a premalignant lesion with malignant potential, adds further complexity to management. We report a woman in her late 40s with uterine didelphys who had persistent atypical endometrial hyperplasia in one uterus and hyperplasia without atypia in the other despite a hormonal therapy trial. Preoperative imaging, including renal ultrasonography, excluded associated urological anomalies. Definitive management with total laparoscopic hysterectomy and bilateral salpingo-oophorectomy was performed. Intraoperatively, two uteri and cervices were confirmed. The surgery was technically challenging due to the altered pelvic anatomy; however, meticulous dissection with careful ureteric identification enabled safe mobilisation and vascular control. A McCartney tube was utilised to aid fornix identification, maintain pneumoperitoneum after colpotomy, and facilitate secure laparoscopic vault closure. The patient tolerated the procedure well, recovered uneventfully, and was discharged home in good condition. At the six-week follow-up, she remained well with no postoperative complications. Histopathological examination confirmed atypical hyperplasia confined to one uterine cavity and benign endometrium in the other, with no evidence of malignancy. This case highlights the importance of thorough preoperative planning and precise surgical technique in managing Müllerian anomalies. It also highlights the rare coexistence of dual endometrial pathology in uterine didelphys, failure of conservative therapy. It highlights that definitive management can be safely and effectively achieved using a laparoscopic approach, despite the challenges of complex pelvic anatomy.

## Introduction

Uterine didelphys is a rare Müllerian duct anomaly characterised by complete duplication of the uterus and cervix. It results from the failure of midline fusion of the paramesonephric ducts during embryogenesis [1]. Müllerian anomalies occur in approximately 4-7% of the general female population, with higher prevalence in selected groups such as infertile women 6-7%, and about 13-16% among those with a history of recurrent pregnancy loss [2]. They are classified by the American Society for Reproductive Medicine according to the extent of fusion and resorption defects, with uterine didelphys representing a complete fusion anomaly [3]. While many women with this condition remain asymptomatic, it is sometimes associated with menstrual irregularities, reproductive challenges, or anomalous pelvic anatomy that complicates surgical procedures; duplication of the uterine corpus and cervices can laterally displace the uterine arteries and ureters, increasing intraoperative risk such as vascular or ureteric injuries [4]. Endometrial hyperplasia with or without atypia can be managed initially with progestin therapy to reverse the pathology and lower malignant potential changes. However, atypical hyperplasia carries a higher risk of progression to endometrial carcinoma. Therefore, definitive surgical intervention is necessary when conservative management fails or patients prefer surgical treatment. The presence of this condition in the context of uterine didelphys is exceptionally rare and necessitates definitive surgical management [5].

Laparoscopic hysterectomy is widely accepted for treating premalignant endometrial disease; however, its application in the context of duplicated uterine anatomy presents unique technical obstacles such as distorted landmarks and increased risk of ureteral or vascular injury [6]. Adjuncts such as the McCartney tube, a transvaginal device that outlines the vaginal fornix and maintains pneumoperitoneum after colpotomy, can enhance safety during dissection and vault closure in complex laparoscopic procedures. Recent evidence comparing different methods of laparoscopic vaginal cuff management highlights the value of thoughtful intraoperative planning and appropriate adjunct use [7].

We report a rare presentation of uterine didelphys with dual-pattern endometrial hyperplasia refractory to hormonal therapy, managed successfully with total laparoscopic hysterectomy and bilateral salpingo-oophorectomy, where meticulous preoperative planning and intraoperative technique, including the strategic use of a McCartney tube, allowed safe completion of the procedure. This report illustrates how such adaptations can overcome anatomical complexity and support effective surgical outcomes.

## Case presentation

A perimenopausal nulliparous woman with a history of two miscarriages and a known diagnosis of uterine didelphys presented with long-standing irregular and heavy menstrual bleeding that had progressively affected her quality of life. Her condition was incidentally diagnosed in early adulthood during evaluation for an abnormal cervical smear, which revealed a double vagina and double cervix; later, she was confirmed to have uterine didelphys. At that time, she underwent vaginal septum excision for dyspareunia and difficulties using tampons. Initial management with pharmacological agents, including mefenamic acid, tranexamic acid, and progestogen-only pills (minipills), provided only limited benefit. Subsequently, she trialled a levonorgestrel-releasing intrauterine system (Mirena®) to control her symptoms.

Office hysteroscopy initially showed two narrow cervices and two narrow uterine cavities with otherwise normal-appearing endometrium; biopsies were obtained. Transvaginal ultrasonography demonstrated uterine duplication with variable endometrial thickness over time: an earlier scan recorded right 7.0 mm and left 7.1 mm, while a later scan recorded right 7.0 mm and left 2.3 mm.

Further evaluation with hysteroscopy and directed endometrial biopsies demonstrated persistent atypical endometrial hyperplasia in one uterine cavity and endometrial hyperplasia without atypia in the contralateral uterus. Given the malignant potential of atypical endometrial hyperplasia and the failure of conservative management with a Mirena device for nearly one year, definitive surgical treatment was recommended after review and detailed patient counselling.

The patient underwent a total laparoscopic hysterectomy with bilateral salpingo-oophorectomy. Preoperatively, renal ultrasonography was undertaken to exclude associated urological anomalies; no abnormalities were identified. Intraoperatively, two separate uterine bodies and two cervices were confirmed. A standard four-port laparoscopic technique was employed. Because of the complex anatomy, a McCartney tube was inserted vaginally to aid fornix identification, maintain pneumoperitoneum following colpotomy, and facilitate secure closure of the vaginal vault (Video 1).

**Video 1:** Steps of the total laparoscopic hysterectomy with bilateral salpingo-oophorectomy in a patient with uterine didelphys The video demonstrates the use of a McCartney tube to aid fornix identification, maintain pneumoperitoneum after colpotomy, and facilitate secure laparoscopic vault closure.

Each hemi-uterus and adnexa were mobilised with meticulous dissection and bilateral ureteric identification. The infundibulopelvic and round ligaments were coagulated and transected individually. Broad ligaments were opened, and the uterine arteries were skeletonised and secured bilaterally using a LigaSure device. The median raphe between the two cervices was carefully dissected, followed by circumferential colpotomy around both cervices under McCartney tube guidance. Both uteri and adnexa were removed vaginally, and the vaginal vault was closed laparoscopically with a continuous barbed V-Loc suture, achieving good approximation and haemostasis. Estimated blood loss was about 100 mL, and no intraoperative complications occurred.

The patient tolerated the procedure well, with an uneventful postoperative recovery, and was discharged on day two after surgery.

Histopathological examination of the specimen comprised two uteri with two cervices, and bilateral adnexa reveals that one uterine cavity showed residual atypical endometrial hyperplasia with treatment effect; the contralateral cavity showed benign endometrium without atypia or malignancy. The myometrium contained adenomyosis and a small leiomyoma. Both ovaries revealed occasional benign follicular/functional cysts and were free of neoplasia. Cervices, parametria, and fallopian tubes were unremarkable (Table 1).

**Table 1:** Summary of histopathological findings

Specimen/Site	Macroscopic Description	Microscopic Description
Right uterus/Cervix	External os 1.1 cm; endocervix normal. Endometrium up to 0.4 cm thick with focal irregular narrowing. Myometrium has a uniform thickness of 1.3 cm, containing a 1.0 cm fibroid	Endometrial cavity shows good progestogenic effect consistent with the presence of Mirena® intrauterine system (IUS). Myometrium shows adenomyosis and benign leiomyoma; no malignancy. No evidence of atypia, hyperplasia, or malignancy was identified
Left uterus/Cervix	External os 1.2 cm; endocervix normal. The endometrium is up to 0.5 cm thick and irregular, with Mirena® in situ. Myometrium normal	The endometrial cavity demonstrates modified tall columnar and papillary glands exhibiting progesterone-related changes. Focal gland crowding and cytological atypia consistent with atypical hyperplasia. No malignancy detected
Right and left cervices	Normal appearance with intact external os	Show reactive epithelial features only; no dysplasia or malignancy identified
Right fallopian tube	5.5 cm long with normal fimbriae and serosa	No significant histopathological abnormality
Left fallopian tube	5.5 cm long with normal fimbriae and serosa	No significant histopathological abnormality
Right ovary	4.0 × 2.3 × 1.7 cm containing serous cysts up to 2.5 cm	Benign follicular and functional cysts; no evidence of malignancy
Left ovary	3.1 × 2.0 × 1.7 cm with serous cysts up to 1.6 cm	Benign follicular and functional cysts; no evidence of malignancy

At six weeks postoperatively, the patient was well with no urinary, bowel, or vault-related complications and was started on hormone replacement therapy for surgical menopause. At six months, she remained asymptomatic, with no late complications, and her menopausal symptoms were effectively managed with ongoing hormone therapy in line with standard care.

Table 2 summarises the chronological clinical course, diagnostic findings, and key management interventions in this patient.

**Table 2:** Summary of the clinical course, interventions, and key findings

Phase	Event/Assessment	Findings or Interventions	Outcome
Background and History	Nulliparous, Known uterine didelphys; history of two miscarriages; prior vaginal septum excision	Condition incidentally diagnosed in early adulthood during evaluation for abnormal cervical cytology, revealing double cervices and vagina; septum excised for dyspareunia and tampon difficulties	Restored vaginal anatomy
Initial Presentation and Medical Management	Perimenopausal with long-standing irregular and heavy menstrual bleeding	Symptoms progressively affect the quality of life despite pharmacological therapy with mefenamic acid, tranexamic acid, and progestogen-only pills	Inadequate control of symptoms. Required medical assessment and symptomatic management
Diagnostic Evaluation and Hormonal Therapy	Levonorgestrel-releasing intrauterine system (Mirena®) used for approximately 12 months. Office hysteroscopy and endometrial biopsies	Persistent abnormal bleeding. Hysteroscopy: Two narrow cervices and uterine cavities with normal-appearing endometrium; biopsies showed atypical endometrial hyperplasia in one uterus and non-atypical hyperplasia in the other	Dual endometrial pathology confirmed and consider conservative management
Reassessment	Repeat hysteroscopy and targeted	Persistent atypical endometrial hyperplasia in one uterine cavity	Failed conservative therapy; indication for definitive surgical management: given the malignant potential
Preoperative Imaging	Transvaginal and renal ultrasonography	Demonstrated double uteri no urological anomalies detected	For definitive surgical intervention
Definitive Surgery	Total laparoscopic hysterectomy with bilateral salpingo-oophorectomy	The McCartney tube used for fornix delineation, pneumoperitoneum maintenance, and secure laparoscopic vault closure	Uneventful procedure, Estimated blood loss ≈ 100 mL; no intraoperative complications
Histopathology	Two uteri, two cervices, and bilateral adnexa	Residual atypical endometrial hyperplasia with treatment effect in one uterus; benign endometrium without atypia in the other; adenomyosis, small leiomyoma, and benign ovaries with follicular cysts	No evidence of malignancy
Postoperative Course	Inpatient recovery	Tolerated procedure well; discharged on postoperative day 2	Uneventful postoperative recovery
Follow-up	Six-week and six-month reviews	No urinary, bowel, or vault-related complications; commenced on hormone replacement therapy for surgical menopause	Well and asymptomatic; menopausal symptoms effectively managed

Table 3 summarises the preoperative and postoperative findings for both uteri and adnexa.

**Table 3:** Summary of preoperative and postoperative findings for both uteri and adnexa

Uterus	Pre-operative Assessment	Histopathology (Post-operative)	Interpretation/Outcome
Right uterus	Hysteroscopy and biopsy showed atypical endometrial hyperplasia; endometrium thickened (≈7 mm on ultrasound)	Residual atypical endometrial hyperplasia with progestin-induced treatment effect; no carcinoma	Persistent premalignant change despite hormonal therapy → indication for definitive surgery
Left uterus	Hysteroscopy and biopsy revealed endometrial hyperplasia without atypia; endometrium variable thickness (7 → 2 mm)	Benign endometrium without atypia or malignancy	Complete regression with medical therapy; no residual abnormality
Myometrium	Not specifically abnormal on imaging	Adenomyosis and small leiomyoma	Benign co-existing pathology
Adnexa (bilateral)	Normal on ultrasound	Benign functional follicular cysts; no neoplasia	No malignant features
Cervices/Parametria/Tubes	Two narrow cervices; normal appearance	Unremarkable histology	No dysplasia or malignancy

## Discussion

Uterine didelphys is an uncommon congenital Müllerian anomaly arising from incomplete fusion of the paramesonephric ducts during embryogenesis [8]. It is estimated to account for around 8% of all uterine malformations [9]. While many women remain asymptomatic, some may present with abnormal uterine bleeding, pelvic pain, subfertility, or obstetric complications [10]. Given the premalignant potential of atypical hyperplasia, definitive surgical intervention is warranted when conservative management fails.

Hysterectomy in duplicated uterine anatomy carries distinct risks. Displacement of the uterine arteries and ureters, fused or attenuated peritoneal reflections, and altered tissue planes can increase the likelihood of ureteric or vascular injury [6]. Different approaches to hysterectomies have been described, but laparoscopic hysterectomies are increasingly favoured due to magnified visualisation, lower morbidity, and faster recovery [11]. Nonetheless, such procedures require preoperative planning, meticulous intraoperative dissection, and thoughtful intraoperative adaptation.

In our case, total laparoscopic hysterectomy with bilateral salpingo-oophorectomy was safely performed despite several anatomic challenges. There was a distortion of the midline pelvic anatomy with the presence of the median raphe. In addition, the broad, irregular configuration of the vaginal vault adds further difficulty to colpotomy and vault closure. In some cases, the uterine arteries and veins may follow atypical courses, and the ureters are displaced, increasing the risk of injury during dissection. The intraoperative use of a McCartney tube proved particularly advantageous, facilitating fornix identification, maintaining pneumoperitoneum after colpotomy, and supporting secure vault closure. While this device has been described in routine laparoscopic hysterectomy [12], its application in congenital anomalies such as uterine didelphys has rarely been highlighted, underscoring its potential as a valuable adjunct in complex anatomy. The intra- and postoperative course was favourable, and the patient remained well at six-week and six-month follow-up. Histopathology result demonstrated residual atypical endometrial hyperplasia with treatment effect confined to one uterus and benign endometrium in the contralateral uterus, with no invasive malignancy.

This case underscores several key learning points. Comprehensive preoperative imaging, including renal tract assessment, is essential, as genitourinary anomalies frequently coexist with Müllerian malformations [13]. Intraoperative identification of the ureters and careful staging of vascular control are critical to minimising complications. Finally, adjuncts such as the McCartney tube can enhance surgical safety and efficiency in challenging operative scenarios.

The limitation of this report is that it is a single case report and it is less generalisable. However, it adds to the scarce body of literature reporting laparoscopic hysterectomy in patients with uterine didelphys and premalignant endometrial pathology and provides a useful intraoperative technique in the management of such cases.

## Conclusions

This case demonstrates that total laparoscopic hysterectomy with bilateral salpingo-oophorectomy can be performed safely and effectively in a patient with uterine didelphys and coexisting premalignant endometrial pathology when meticulous preoperative planning and precise surgical execution are applied. The strategic use of a McCartney tube proved invaluable in navigating duplicated pelvic anatomy, facilitating accurate dissection, maintaining pneumoperitoneum, and ensuring secure vault closure.

The findings are not generalisable as a single case. Further case accrual and multicentre experience are needed to confirm reproducibility, refine technical steps, and define when adjuncts such as the McCartney tube add measurable benefit in minimally invasive management of complex Müllerian anomalies.
